# Dentin Grafts: Navigating the Paradigm Shift in Regenerative Dentistry

**DOI:** 10.7759/cureus.70760

**Published:** 2024-10-03

**Authors:** Divya Suvarna Dixit, Bhushan P Mundada, Nitin Bhola, Anchal Agarwal

**Affiliations:** 1 Oral and Maxillofacial Surgery, Sharad Pawar Dental College and Hospital, Datta Meghe Institute of Higher Education & Research, Wardha, IND

**Keywords:** biocompatibility, dentin grafts, osteoinductive potential, regenerative dentistry, tissue regeneration

## Abstract

The biomaterial of dentin has emerged as a promising candidate for the tissue engineering of dental hard tissues. In bone tissue engineering, it may serve as either a scaffold or a reservoir of growth factors. The physical and chemical similarities between the dentin structure and bone have sparked scientific interest in using its features for the development of a new bone transplant material. Dentin, unlike hard and fragile enamel, is viscoelastic, making it a very effective bone replacement. The regeneration of pulp tissue has proven challenging due to its encasement in dentin, which lacks collateral blood flow except from the apical end of the root. Yet, the emergence of contemporary tissue engineering and the identification of dental stem cells have enabled experimentation with the regeneration of both pulp and dentin. This review will explain the different types of dentin grafts, their biocompatibility, safety, and effectiveness, along with difficulties. Additionally, the paper covers several strategies for creating autogenous dentin grafts and gives evidence-based insights into their clinical effectiveness. Overall, dentin grafts appear as a potential alternative to standard graft materials, stimulating tissue regeneration and enhancing patient outcomes in regenerative dentistry operations.

## Introduction and background

Despite major breakthroughs in dentistry, oral and dental disorders remain a vital global worry. Oral and dental treatments often use synthetic materials that possess characteristics differing from those of natural tissues, leading to their eventual failure even under optimal settings [1]. Tissue engineering is a compelling subject within the fields of regenerative medicine and dentistry. Tissue engineering, using a combination of stem cells, scaffolds, and growth factors, offers favorable prospects for the regeneration of damaged or absent tissues. Dentin is a potential biomaterial for tissue engineering, functioning as both a scaffold and a substantial source of growth factors. Dentin is a calcified connective tissue, and its properties are mostly determined by its mineralized extracellular matrix. This tissue has 50% minerals, 30% organic molecules, and 20% water [2]. However, the distribution of these components varies among several regions and types of dentin. Various clinical uses of dentin graft are shown in Table 1.

**Table 1:** Clinical application

Socket preservation
Periodontal defects and regeneration
Ridge augmentation
Sinus lift procedures
Alveolar cleft reconstruction
Implant site preparation
Root canal filling material
Trauma and defect repair

In this study, we aim to explore dentin grafts, covering their history, properties, preparation, and applications. By examining current literature and recent advancements, we will assess their compatibility, effectiveness, and potential as a preferred option for dental treatments. Additionally, this review will address the challenges associated with their implantation and discuss their future potential.

## Review

**Figure 1:**
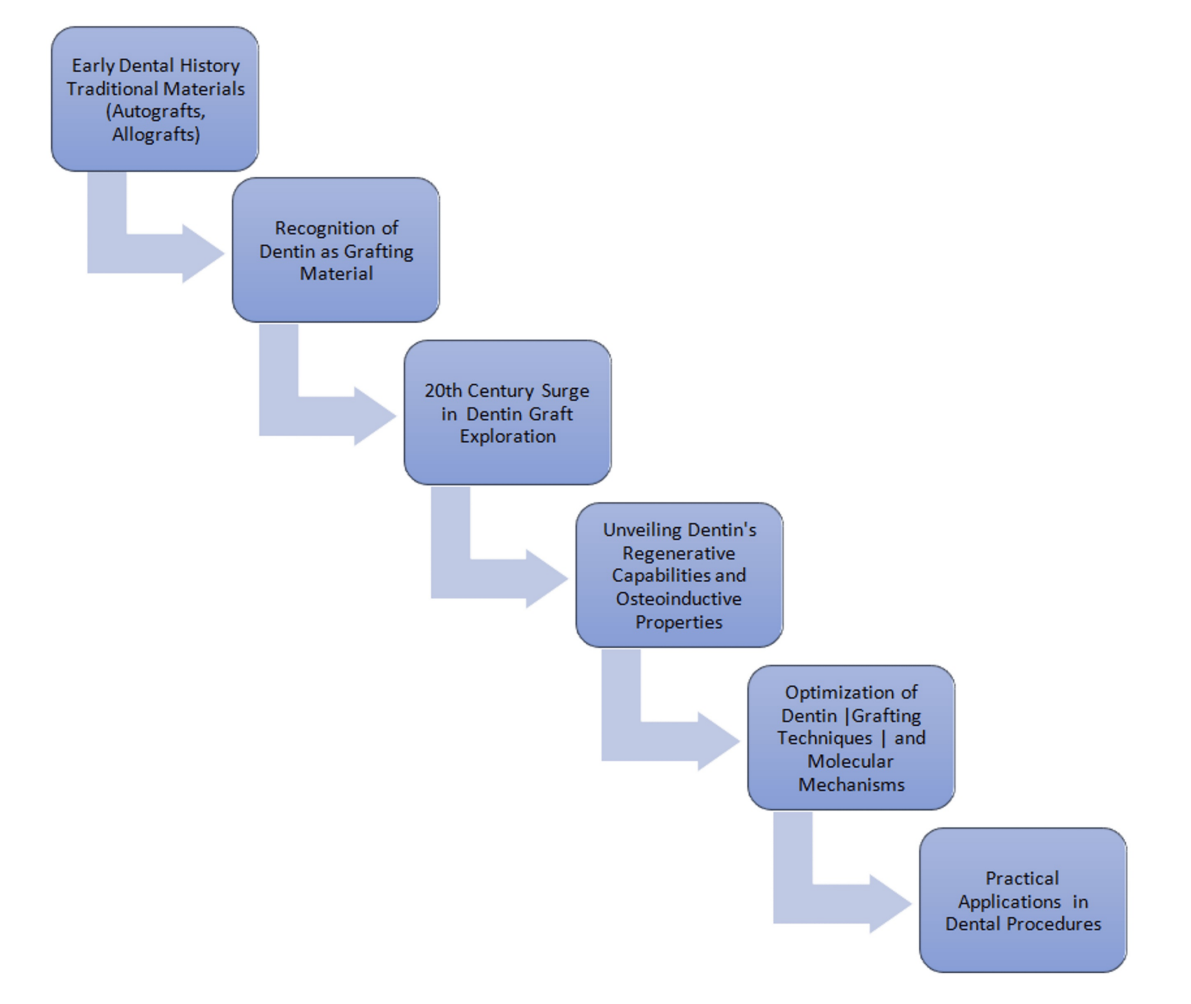
Evolution of dentin as a graft material Image credit: Divya Suvarna Dixit

Even with significant advancements in dentistry, oral and dental illnesses continue to be a serious worldwide issue. Dental and oral treatments are often administered using artificial materials that differ from real tissues in some ways, and as a result, even under the best of circumstances, they eventually fall short. A novel concept in dentistry and regenerative medicine is tissue engineering. Tissue engineering, which combines scaffolds, growth factors, and stem cells, offers hope for the regeneration of missing or damaged tissues. Dentin is a biomaterial that shows promise for tissue engineering applications since it may function as a scaffold and a rich source of growth factors [3]. The mineralized extracellular matrix of dentin, a calcified connective tissue, is essentially responsible for its properties. Twenty percent is water, 30% is biological matter, and 50% is minerals. The distribution of these elements varies throughout dentin types and distinct parts, nevertheless. The era marked a transition from theoretical speculation to practical applications, with dental practitioners incorporating dentin grafts in diverse procedures, from periodontal treatments to implant surgeries, as shown in Figure 1.

The dentin matrix’s organic composition consists of several connective tissue-specific macromolecules. This matrix also includes substances that are exclusive to mineralized tissues. The matrix is produced by odontoblasts and is a rich source of bioactive chemicals and growth factors needed for dentinogenesis. These substances are released in the event of dental caries or certain materials used in restorative procedures, and they promote dentin regeneration and repair. Ethylenediaminetetraacetic acid etchants produce dentinal matrix chemicals that exhibit strong morphogenetic activity and incite dentinogenesis in vivo. Noncollagenous and collagenous proteins, proteoglycans, glycoproteins, and lipids are all found in the dentin organic matrix. Ninety percent of the dentin extracellular matrix proteins are collagens, and type I collagen, which makes up most of the bone, is the most common kind of collagen [4]. In addition to containing several growth factors, including transforming growth factor-b, insulin-like growth factor, bone morphogenetic proteins, and certain angiogenic growth factors, dentin collagen provides a tight and crosslinked framework in which mineral crystals deposit [5]. These growth factors cause dentin disintegration and promote reparative dentinogenesis when they are released concurrently with certain events, such as the formation of cavities.

Sources of dentin graft material might vary, and recognizing the sources is vital for its effective application in different dental operations. Common sources of dentin graft material include autogenous dentin grafts, produced from the patient’s own removed teeth, commonly during regular dental operations or extractions [6]. These autogenous grafts diminish the likelihood of immunological rejection and disease transmission [7]. Another source is dentin from excised teeth, such as wisdom teeth, which repurposes native tissue and reduces the need for repeated harvesting operations [8]. Various forms of dentin grafts are presented in Table 2.

**Table 2:** Sources of dentin graft material

Source	Description	Advantages	Disadvantages
Autogenous dentin grafts	Derived from the patient’s own extracted teeth, minimizing the risk of immune rejection and transmission	Potential for better integration and acceptance by the host tissue	Limited availability and quantity of graft material
Allogenic dentin grafts	Harvested from another individual of the same species, requiring proper processing to reduce risks	Can provide a viable graft source when autogenous grafts are not feasible	Risk of immune rejection and disease transmission, necessitating thorough processing
Xenogenic dentin grafts	Obtained from a different species, typically porcine or bovine, with rigorous processing for safety	Potential for reduced patient morbidity as no second surgical site is required	Risk of immune response due to interspecies differences
Decellularized dentin matrices	Dentin matrices with removed cellular components retain structure while minimizing immunogenicity	Provides a scaffold for tissue regeneration without the risk of disease transmission	Complex processing methods are needed to remove cellular components
Synthetic dentin substitutes	Lab-engineered materials mimicking natural dentin properties, often based on hydroxyapatite or ceramics	Eliminates the risk of disease transmission and immune rejection	Long-term stability and integration with surrounding tissues may be a concern
Dentin from extracted teeth	Utilizing extracted teeth, such as wisdom teeth, as a source of graft material, repurposing natural tissue	Utilizes a readily available source without the need for an extra surgical procedure	Potential for variations in the quality and quantity of graft material

In dental grafting, the choice between dentin grafts and traditional bone grafts is a critical decision that clinicians must make based on various factors. This comparative analysis aims to highlight the distinctions between these two approaches, shedding light on their advantages, limitations, and specific clinical considerations [9]. These factors are discussed in Table 3.

**Table 3:** Comparative analysis of various factors in dentin grafts and traditional bone grafts

Properties	Dentin graft	Traditional bone graft
Biological properties	Exhibit osteoinductive properties, promoting natural bone regeneration due to the presence of growth factors	Provide a scaffold for bone formation, lacking the inherent biological signaling seen in dentin
Osteoinductive potential	Known for their intrinsic ability to stimulate the surrounding cells and tissues for enhanced bone formation	Rely on the structural support provided by the graft, requiring a longer integration period
Biocompatibility	Generally well-tolerated by the host, reducing the risk of rejection	May pose a higher risk of immune response, especially with allogenic or xenogenic sources
Clinical applications	Widely used in various dental procedures, including socket preservation, periodontal defects, and implant placements	Applied in a range of procedures, such as ridge augmentation, sinus lifts, and complex implant cases
Harvesting complexity	Harvesting is typically less invasive, especially when utilizing extracted teeth or commercially available dentin products	Autogenous bone harvesting can be more invasive, requiring an additional surgical site
Availability and cost	Can be sourced from readily available extracted teeth, potentially reducing costs	Availability may vary, and autogenous grafts may involve higher costs and additional patient discomfort
Limitation	The need for additional personnel to assist with dentin graft preparation and chemical processing is a limitation of the chairside approach	Traditional bone grafting carries inherent risks of complications such as infection, graft rejection, or inadequate bone integration

**Figure 2:**
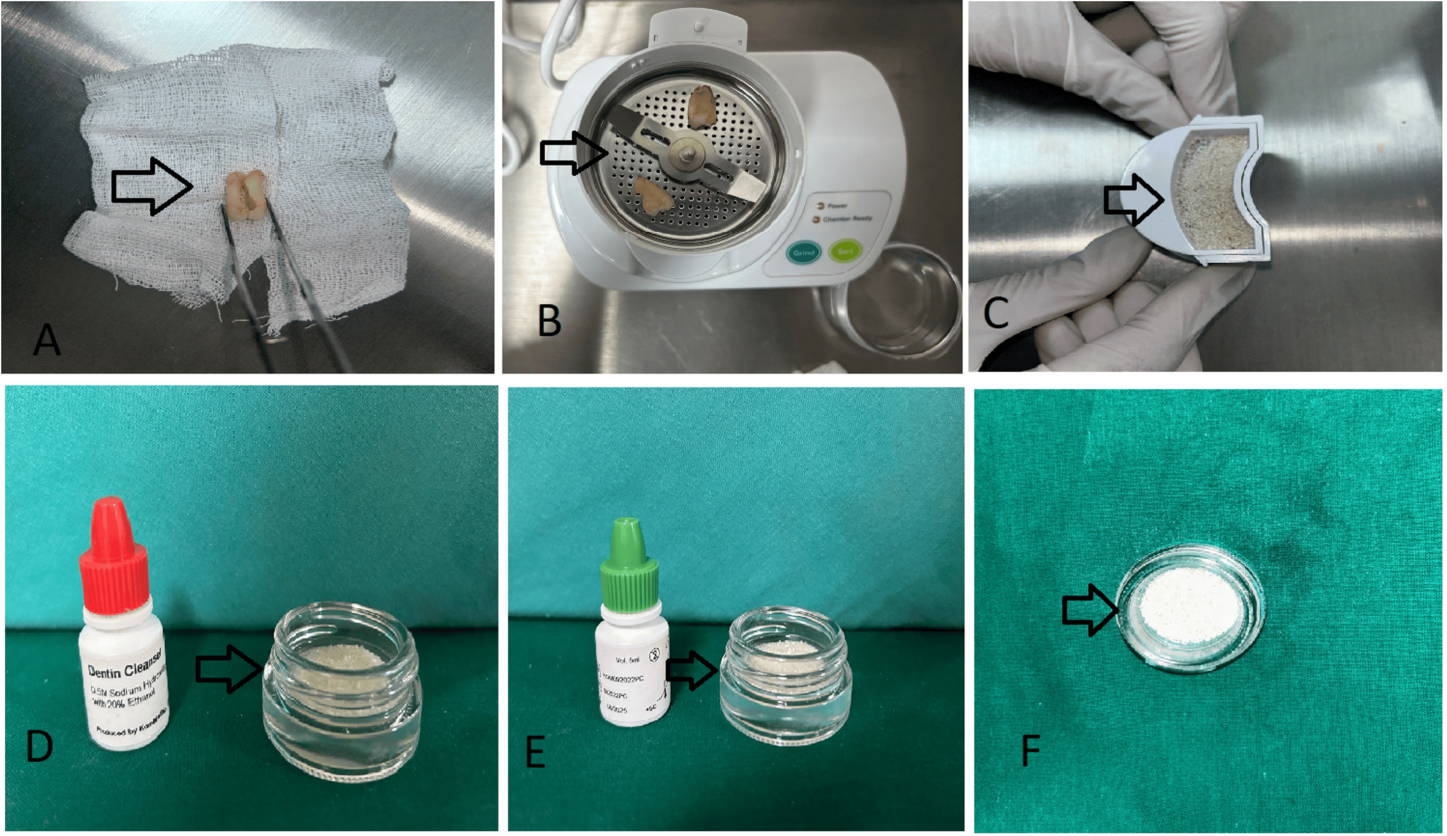
Preparation of an autogenous dentin graft (A) Extracted tooth. (B) Extracted tooth in dentin grinder. (C) Dentin graft. (D) Cleaning of dentin graft. (E) Dentin graft processed. (F) Dentin graft prepared. Image credit: Divya Suvarna Dixit

Dentin grafts have emerged as a promising avenue in regenerative dentistry, and evaluating their biocompatibility and safety is paramount for successful clinical implementation [10]. Extensive research literature provides compelling evidence supporting the favorable biocompatibility profile of dentin grafts, especially when sourced from the patient’s own teeth (autogenous). A 2021 study reports that dentin grafts are generally well-tolerated by the host, exhibiting minimal immunogenic responses and inflammatory reactions. Furthermore, it also conducts comparative analyses against traditional bone graft materials, revealing dentin’s comparable or even superior biocompatibility [11]. Long-term clinical studies have demonstrated stable outcomes and high patient satisfaction rates, affirming the safety of dentin grafts in supporting and regenerating natural bone structures [11]. In 2023, research stressed the necessity of risk mitigation techniques, including correct processing procedures and adherence to regulatory criteria, which play a key role in boosting the overall biocompatibility and safety of dentin transplants [10]. Collectively, the evidence-based insights from scientific literature underline dentin grafts as a dependable and safe alternative for numerous regenerative dental operations. With their high biocompatibility, few adverse responses, and established clinical results, dentin grafts have emerged as a viable alternative to standard graft materials in stimulating tissue regeneration and improving patient outcomes. The process of creating autogenous dentin grafts may be classified into three primary categories: mineralized, partly demineralized, and demineralized. The standard protocol entails several steps. First, the extracted tooth is cleaned and dried. Then, it is ground using a dentin grinding device to achieve a particle size of 300-1,200 μm. Next, it is treated with a solution of 0.5 molar sodium hydroxide and 20% ethanol for 12 minutes to remove any organic remains, bacteria, or toxins. Finally, the tooth is submerged in saline for three minutes, as depicted in Figure 2 [12].

By employing just pure alcohol and sodium bicarbonate for disinfection, the mineralized dentine graft preparation method minimizes chemical treatment and maintains the tooth graft’s mineral makeup [13]. By exposing the dentine particles to diluted acids, partial demineralization occurs, removing a portion of the mineral content. This process exposes the organic dentine matrix, which contains bone-regulating proteins such as osteocalcin and osteonectin. To achieve complete demineralization, the dentine graft must be exposed to diluted acids for an extended period of time under vacuum conditions in order to remove the majority of the inorganic components, leaving behind just the organic dentine matrix [14]. The graft particles may be used as a foundation for bioengineering either by themselves or in combination with other materials, including injectable platelet-rich fibrin [15].

A search was conducted in PubMed for studies using an autogenous dentin graft prepared from an extracted tooth using a dentin grinder. The following keywords were used: “autogenous dentin graft” or dentin graft in dentistry. After examining the title and abstract, if we did not find enough information, we obtained the full text to evaluate the study’s validity for inclusion in our review. After a comprehensive screening process, a total of 25 articles were selected from an initial pool of 46 articles. The studies that met the eligibility criteria were chosen for inclusion in our review. A summary of these selected studies is presented in Table 4.

**Table 4:** Comparison of studies by various authors

Year	Author	Types of graft used	Aim of study	Parameters of study
2021	Cervera-Maillo et al. [2]	Autologous dentin graft	Using removed teeth that are ground into bacterial-free dentin fragments using a Smart Dentin Grinder and promptly transplanted into the alveolus after extraction or into bone defects, assess the effectiveness of an autologous dentin graft	Post-extraction sockets and implant gap grafted with dentin graft material, and natural healing sockets, in all cases used in the immediate post-extraction implant protocol
2020	Sánchez-Labrador et al. [11]	Autogenous dentin graft	Assessment of autogenous dentin transplant in cases with bone deficiency after excision of the lower third molar (a split-mouth clinical)	Measuring the depth of the probing at three and six months after surgery
2021	Um et al. [15]	Autogenous demineralized dentin matrix	The application of dentin graft material from other individuals — allogeneic DDM — has been considered as an alternative to auto-DDM	Steoinductivity and antigenicity of allo-DDM allogeneic dentin application for the management of maxillofacial bone defects
2015	Kim [16]	Autogenous fresh demineralized tooth graft	Analyzing the clinical utility of chairside-prepared autogenous fresh demineralized tooth grafts for alveolar bone grafting in dental implant surgery	Clinical findings, implant success rate, and histological evaluation
2015	Kim et al. [17]	Autogenous fresh demineralized tooth (block, chip, or powder)	Evaluation of chairside preparation of autogenous fresh demineralized teeth for socket preservation right after extraction in a clinical setting	Radiographic evaluation and histological evaluation of two samples
2017	El-Said et al. [18]	Autogenous fresh demineralized tooth graft	Assessing the effectiveness of chairside-prepared autogenous fresh demineralized tooth grafts for alveolar bone grafting in newly removed sockets in dental implant surgery	Clinical evaluation, radiological evaluation, and histological evaluation
2019	Fathy et al. [19]	Mineralized dentin particulate graft	An evaluation of the effects of socket preservation using mineralized dentin particle graft	Cone beam CT images were used to compare horizontal and vertical ridge diameters and bone density readings immediately post-surgical and six months after extraction
2019	Upadhyay et al. [20]	An autogenous tooth transplant made from a recently removed tooth is produced chairside	Clinical assessment of autogenous tooth graft as a new bone graft material in the treatment of Class II furcation problems	Mean reductions in horizontal probing depth and mean gains in linear bone fill
2019	Minetti et al. [21]	Extracted teeth mixed with an equal quantity of xenograft	Compare the histological results after socket preservation between dentin mixed with xenograft and dentin alone in tooth graft procedure	Three walls of post-extractive defects requiring the restoration of bone dimension and shape in the mandibular zone
2019	Wu et al. [22]	Autogenous tooth bone produced from the removed teeth by chair-side	Comparison of the efficiency of the autogenous tooth bone and xenogenic bone grafted in rapid implant placement in front teeth with a bone deficit	Clinical examination and radiographic examination of the horizontal bone change in the level of 0 mm, 3 mm, and 6mm below the implant neck and the marginal bone loss immediately, six, and 12 months after implant insertion. Questionnaire about the sentiments regarding the operation at the moment of removing the sutures
2020	Kuperschlag et al. [23]	Autogenous dentin graft	Evaluation of osseous healing after surgical excision of impacted mesioangularly or horizontally inclined third molars using the processed third molar as the graft material, followed by guided bone regeneration treatment of osseous defects distal to mandibular second molars	Clinical and radiological examinations, including panoramic radiographs and probing depths at three months and 12 months postoperatively
2020	Dwivedi and Kour [24]	Autogenous fresh mineralized tooth graft prepared at the chairside	Alveolar ridge preservation using autogenous fresh mineralized tooth graft prepared at chairside	Using a three-dimensional imaging method, the radiographic assessment of alveolar ridge preservation was carried out
2021	Yüceer-Çetiner et al. [25]	Autogenous dentin graft	Examination of the effects on bone healing processes of autogenous dentin graft and combination of autogenous dentin graft and platelet-rich fibrin injected into tooth extraction sockets	After three months, histological and immunohistochemical evaluations on the samples taken during the implant surgery. Samples obtained from each group were examined by scanning electron microscopy
2022	Gabr et al. [26]	Autogenous fresh tooth graft	Comparative analysis of autogenous fresh tooth grafts around immediate dental implants with and without platelet-rich fibrin	Peri-implant probing depth, implant stability, and radiographic evaluation of vertical and horizontal dimensional changes
2022	Shoeib et al. [27] (preprint)	Demineralized dentin particles	Contrasting the xenograft in thin buccal bone with the initial appearance of soft tissue around dental implants using dentin chips	Pink aesthetic scores after a year after implant implantation and after loading at six months. Using CBCT, the resorption of buccal and crestal bone was assessed at six and one year. Measured before loading and just after implant implantation is implant stability
2022	Gupta et al. [28]	Processed dentin particulate graft	Comparison of the efficiency of the dentin autograft with autogenous bone graft for maintenance of socket defect following removal of mandibular third teeth	Clinical and radiographic evaluation
2022	Minetti et al. [29]	Autogenous dentin particulate graft	Histological evaluation of bone after autogenous dentin particle grafting with and without a resorbable collagen membrane for alveolar ridge enhancement	Histomorphometric analysis
2022	Mazzucchi et al. [30]	Autologous dentin graft	Radiographic and periodontal assessment of post-extractive sockets	Radiographic and periodontal evaluation of post-extractive sockets
2023	Abo-El-Saad et al. [31]	Autogenous demineralized dentin graft	Comparison of alloplastic and autogenous dentin grafts combined with socket shields for the preservation of pre-implant sockets	Histological analysis, histomorphometric analysis, and radiographic analysis
2023	Sapoznikov et al. [32]	Ivory dentin graft	Ivory Dentin Graft™ in comparison with a commercial bone-derived material	Evaluating the clinical safety, tolerability, and performance
2023	Wu et al. [33]	Extrafibrillarly demineralized dentin matrix	Extrafibrillar demineralization is introduced into the construction of dentin-derived biomaterials for bone regeneration	Scaffolds for bone regeneration
2023	Feng et al. [34]	Autogenous particulate dentin	Histomorphometric and clinical efficacy of autogenous particulate dentin in alveolar ridge preservation in comparison to other grafted materials or blood clot healing	Horizontal and vertical dimensions of the extraction socket osteogenic properties degradation capacity

In a study conducted in 2021, a comparison was made between the clinical and histological/histomorphometric outcomes of employing autogenous mineralized dentin grafts and xenograft granules for alveolar ridge preservation following tooth extraction [35]. The clinical observations, including implant stability, demonstrated similarity between both groups. However, when the histomorphometric examination was performed, it was shown that, in contrast to sockets maintained with xenograft, sockets conserved with mineralized dentin matrix showed more freshly produced bone and less leftover graft. Emerging technologies and innovations in dentin grafting include decellularized dentin matrices, which use advanced processing techniques to preserve the extracellular matrix while eliminating cellular components, improving biocompatibility, reducing immunogenicity, and enhancing scaffold properties for tissue regeneration [36]. Three-dimensional printing technology is being utilized to create customized dentin graft scaffolds with precise geometries, allowing for tailored grafts for patient-specific anatomies and promoting optimal integration and functional outcomes [37]. Biomimetic approaches focus on developing materials that closely mimic the composition and properties of natural dentin, enhancing compatibility and regenerative potential [38]. Gene therapy techniques are being explored to stimulate specific molecular pathways for accelerated dentin regeneration, offering targeted and efficient enhancement of regenerative processes and potentially reducing healing times [39]. Smart materials and drug delivery systems are being integrated into dentin grafts for controlled release of growth factors or therapeutic agents, allowing precise modulation of the graft microenvironment and promoting optimized tissue healing [40]. Nanotechnology is being incorporated into dentin grafts for improved mechanical properties and enhanced cellular interactions, increasing strength and bioactivity, and potentially expanding their applications [41]. Virtual planning tools and augmented reality are being implemented in dentin grafting procedures, improving preoperative visualization, enhancing precision, and reducing procedural risks [42]. Finally, regenerative medicine principles and stem cell therapies are being integrated to augment dentin grafting outcomes, stimulating the innate regenerative potential of tissues and leading to more robust and predictable results [43].

## Conclusions

Particulate dentin grafts have to be taken into account as a substitute substance for sinus lifting, split techniques, and socket preservation. After a 24-month assessment period, one of its unique features was its high rate of resorption and noninflammatory bone repair. The open tubes found in dentin particles enabled capillaries to enter and facilitated rapid resorption. The dentin graft’s performance is at least on par with widely utilized xenogeneic or allogenic biomaterials in terms of both clinical and histological aspects. These findings underscore the potential of dentin grafts to revolutionize contemporary dental practices by offering a biocompatible and efficacious alternative to traditional biomaterials. The ability of dentin grafts to seamlessly integrate with the surrounding bone, coupled with their favorable resorption kinetics and absence of inflammatory responses, heralds a significant advancement in dental regenerative therapies. As such, the continued exploration and validation of dentin grafts through rigorous research endeavors hold the key to unlocking their full clinical utility and establishing them as a cornerstone in modern dental grafting protocols.
